# Ultraviolet patterns of flowers revealed in polymer replica – caused by surface architecture

**DOI:** 10.3762/bjnano.10.45

**Published:** 2019-02-13

**Authors:** Anna J Schulte, Matthias Mail, Lisa A Hahn, Wilhelm Barthlott

**Affiliations:** 1 Nees Institute for Biodiversity of Plants, University of Bonn, Venusbergweg 22, D-53115 Bonn, Germany; 2 Fraunhofer Institute for Technological Trend Analyses INT, Appelsgarten 2, D-53879 Euskirchen, Germany; 3 Institute of Crop Science and Resource Conservation (INRES) – Horticultural Science, University of Bonn, Auf dem Hügel 6, D-53121 Bonn, Germany; 4 Institute of Applied Physics (APH), Karlsruhe Institute of Technology (KIT), Wolfgang-Gaede-Straße 1, D-76131 Karlsruhe, Germany

**Keywords:** biomimetics, hierarchical structures, light absorption, light harvesting, light reflection

## Abstract

Angiosperms and their pollinators are adapted in a close co-evolution. For both the plants and pollinators, the functioning of the visual signaling system is highly relevant for survival. As the frequency range of visual perception in many insects extends into the ultraviolet (UV) region, UV-patterns of plants play an important role in the flower–pollinator interaction. It is well known that many flowers contain UV-absorbing pigments in their petal cells, which are localized in vacuoles. However, the contribution of the petal surface microarchitecture to UV-reflection remains uncertain. The correlation between the surface structure and its reflective properties is also relevant for biomimetic applications, for example, in the field of photovoltaics. Based on previous work, we selected three model species with distinct UV-patterns to explore the possible contribution of the surface architecture to the UV-signaling. Using a replication technique, we transferred the petal surface structure onto a transparent polymer. Upon illumination with UV-light, we observed structural-based patterns in the replicas that were surprisingly comparable to those of the original petals. For the first time, this experiment has shown that the parameters of the surface structure lead to an enhancement in the amount of absorbed UV-radiation. Spectrophotometric measurements revealed up to 50% less reflection in the UV-absorbing regions than in the UV-reflecting areas. A comparative characterization of the micromorphology of the UV-reflecting and UV-absorbing areas showed that, in principle, a hierarchical surface structure results in more absorption. Therefore, the results of our experiments demonstrate the structural-based amplification of UV-reflection and provide a starting point for the design of bioinspired antireflective and respectively strongly absorbing surfaces.

## Introduction

The outer epidermal surface of plants, the cuticle, forms the first and crucial boundary to the abiotic environment [1–2]. In most cases, this cuticle is a multifunctional interface. Many of its functions are determined not only by its chemical composition, but also by its surface micro- and nanoarchitecture. Superhydrophobicity [1] is the classical example which can occur even on a chemically hydrophilic surface. Not only is the wettability important, but it must also be considered that the cuticle will interact with many different environmental influences, for example, wetting, contamination, and electromagnetic radiation [3–4] as well. Ultraviolet (UV) radiation in the wavelength range 280–380 nm is particularly crucial for plants, for example, when interacting with pollinators. For more than a hundred years it has been known that there is a diversity of flowers which produce colors not only in the visible part of the spectrum (VIS, 400–700 nm) but also in the UV-regime (280–400 nm) [5]. These UV-patterns can serve as “nectar guides” to attract pollinators such as bees [6–9], but other functions are also known [10]. A particular pattern in which petal bases absorb UV-radiation while the apices reflect UV-radiation is found in many angiosperms [7]. Numerous investigations have shown that these UV-patterns are based on UV-absorbing pigments, especially flavonoids [8,11–12]. These pigments are localized in the vacuoles within the epidermal cells of the absorbing flower areas [13–14]. Therefore, most of the investigations in plants undertaken so far have concentrated on the influence of these pigments on the appearance of UV-patterns. Almost no attention has been given to the possible influence of the hierarchical petal surface microarchitecture. Structural coloration and structure-based reflection of VIS- and UV-radiation is well known in animals such as butterflies, birds or beetles [15–16]. In plants, periodic structures causing iridescence have been found in leaves, petals or seeds [17–19]. Whitney et al. [20] reported, for example, that this iridescence acts as a cue for pollinators and has also assumed such an effect in the UV-range. However, the influence of the plant surface structure – especially papillae – on UV-reflection up to now has not been studied in detail.

Barthlott and Ehler [21] state that convex-shaped cells like papillae are the most common architecture in the surface structure of flowers. The parameters of these cells, such as height, diameter and tip radius, are as diverse as the arrangements of the cuticular foldings on top [21]. Barthlott et al. provided definitions and examples of such surface structures [1,21]. The surfaces of the three species investigated in this work possess papillae with heights between 20 µm and 80 µm and consist of convex-shaped cells. Different studies have found that conical cells occur on 75–80% of investigated angiosperm petals [13,21]. Several studies have discussed the functions of convex-shaped cells and their cuticular foldings [1,22–25], but little is known about the exact surface parameters. Possibly, such papillate cells are able to enhance VIS- and UV-absorption. Exner and Exner [26] proposed that the primary function of the papillated epidermis of petals is to act as a VIS-light-trap for incident light by reducing the specular reflection on the surface. This process is based on multiple reflections between the convex epidermal cells. Bernhard et al. [27] further reported that Exner and Exner had examined the epidermal cells of the petals of pansies and roses and pointed out that because of their cone-shaped cells the total path length of the light passing through the petal is longer as compared to papillate cells. By this, these (pigment filled) cells act as a kind of color filter. Consequently, thicker cells create a longer path length for the light within the petal and thereby increase the chance that the light reaches the pigments. This results in a stronger stimulation of the pigments and thus a higher spectral purity. Exner and Exner [26] suspect that flowers with a saturated color have a better chance of being seen and therefore have higher reproduction success. It can be assumed that conical papillate cells (for the definition see [21]) increase the amount of light absorbed by the petal (light trapping), reaching the pigments in flowers, and by this, the intensification of the petal color is enhanced. Glover and Martin [28–29] worked on the biological relevance of this color intensification and found that in *Antirrhinum majus*, conical petal cells significantly enhance the flower’s chance of being visited by a pollinator. This was also substantial for the attractiveness of flowers for bees which also recognized the UV-patterns [6,30].

Gorton and Vogelmann [31] showed that about 50–75% of the light reflection difference in flowers can be attributed to the structural differences at the petal surfaces. In previous studies, the statements about the influence of the surface structure on the UV-reflectance are contrary [12,31] – Some postulated an influence while others doubt that there is any influence on the UV-pattern by the surface structure. Furthermore, there are hardly any studies were the structure was separated from the pigments. For example, Lee et al. [32] tried to examine the influence of the surface structure on the UV-reflection using replicas of rose petals with altered (shrunken) epidermal cells, which did not provide any information about the real influence of the original surface structures on the reflection of light.

Mechanisms to reduce the reflection of light and to extend the light path length in the material are particularly interesting for several technical applications [33–35]. Today, anti-reflective coatings are used in a broad range of optical applications, and importantly, for solar cells with a low reflectivity and a low scattering angle, meaning that the light moves along a nearly straight path through the material [34–35]. For this reason the results of this study are interesting not only for a basic understanding of the occurrence of UV-patterns in plants, but they also provide new data for the development of technical surfaces with well-defined optical specifications.

For this purpose the UV-reflection of the flowers of approximately 8,000 angiosperms was systematically recorded and published [36–38]. To determine the possible influence of the surface topography on the appearance of UV-patterns, we investigated the interaction between UV-light and the surface structures of three different plant species in this study. To consider several diverse surface structures we chose three species with distinct UV-patterns and different surface structures, for example, those with and without a cuticular folding and papillae with high and low aspect ratios. To specifically analyze the role of the surface structure in these optical processes, we transferred the surface structures into a polymer material by using a two-step molding process. This replication method is described in Koch et al. [39] and is a suitable technique for the transfer of the surface topography of soft and fragile plant material to a rigid material in high precision down to the nanometer scale.

## Results and Discussion

**Figure 1:**
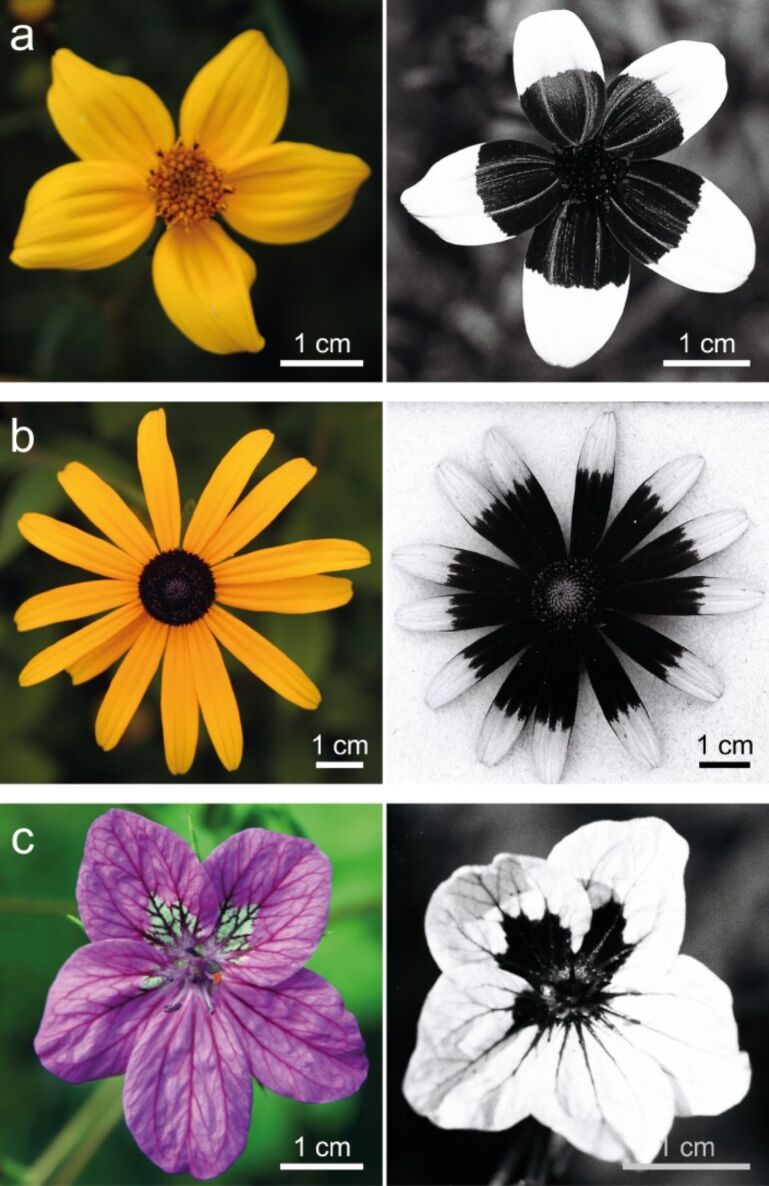
The three model species analyzed in the visible spectrum (left) and UV-regime (right): *Bidens ferulifolia* (a), *Rudbeckia fulgida* (b) and *Erodium manescavii* (c).

Images of flowers under environmental conditions were taken in the VIS- as well as in the UV-range. The images on the left in Figure 1 show flowers of *Bidens ferulifolia* (a), *Rudbeckia fulgida* (b) and *Erodium manescavii* (c) in the VIS-regime, which are the three model species selected for this work. The petals of *B. ferulifolia* and *R. fulgida* are yellow due to the presence of carotenoids, and the *E. manescavii* is purple, due to the presence of anthocyanins. In the images on the right in Figure 1, the flowers of the same species are imaged in the UV-regime. The flowers exhibit UV-patterns where the absorbing area is at the petal base (black area) and the UV-reflecting area is at the petal tip (white area). These UV-patterns are common patterns in angiosperm flowers [7].

**Figure 2:**
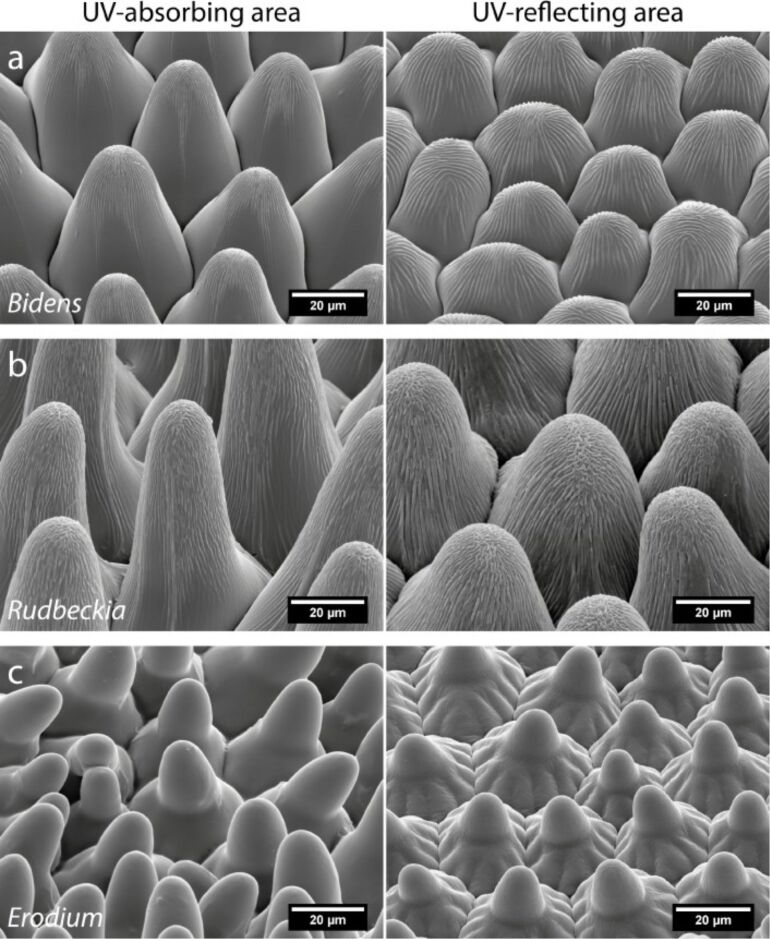
SEM images of the petal surface structure at the UV-absorbing (left) and UV-reflecting (right) areas of *B. ferulifolia* (a), *R. fulgida* (b), and *E. manescavii* (c).

For the analysis of the surface architecture parameters, scanning electron microscopy (SEM) images of the UV-absorbing (Figure 2, left images) and the UV-reflecting (Figure 2, right images) areas of the three model flowers were taken. All petal surfaces possess conical-shaped epidermal cells both in the UV-absorbing and UV-reflecting areas, but differ in their specific characteristics. The papillate epidermal cells in the UV-absorbing areas are higher than in the reflecting areas. *E. manescavii* has no cuticular foldings on top of the papillae. This is in contrast to the papillae of *B. ferulifolia* and *R. fulgida*, which possess cuticular foldings. The measurement of the folds on top of the papillae of *B. ferulifolia* revealed a fold width of about 458.1 ± 93.3 nm (UV-absorbing area) and a fold width in *R. fulgida* of about 539.0 ± 92.8 nm (UV-absorbing area). There were no significant differences between the UV-absorbing and UV-reflecting areas in terms of the cuticular folding width.

**Figure 3:**
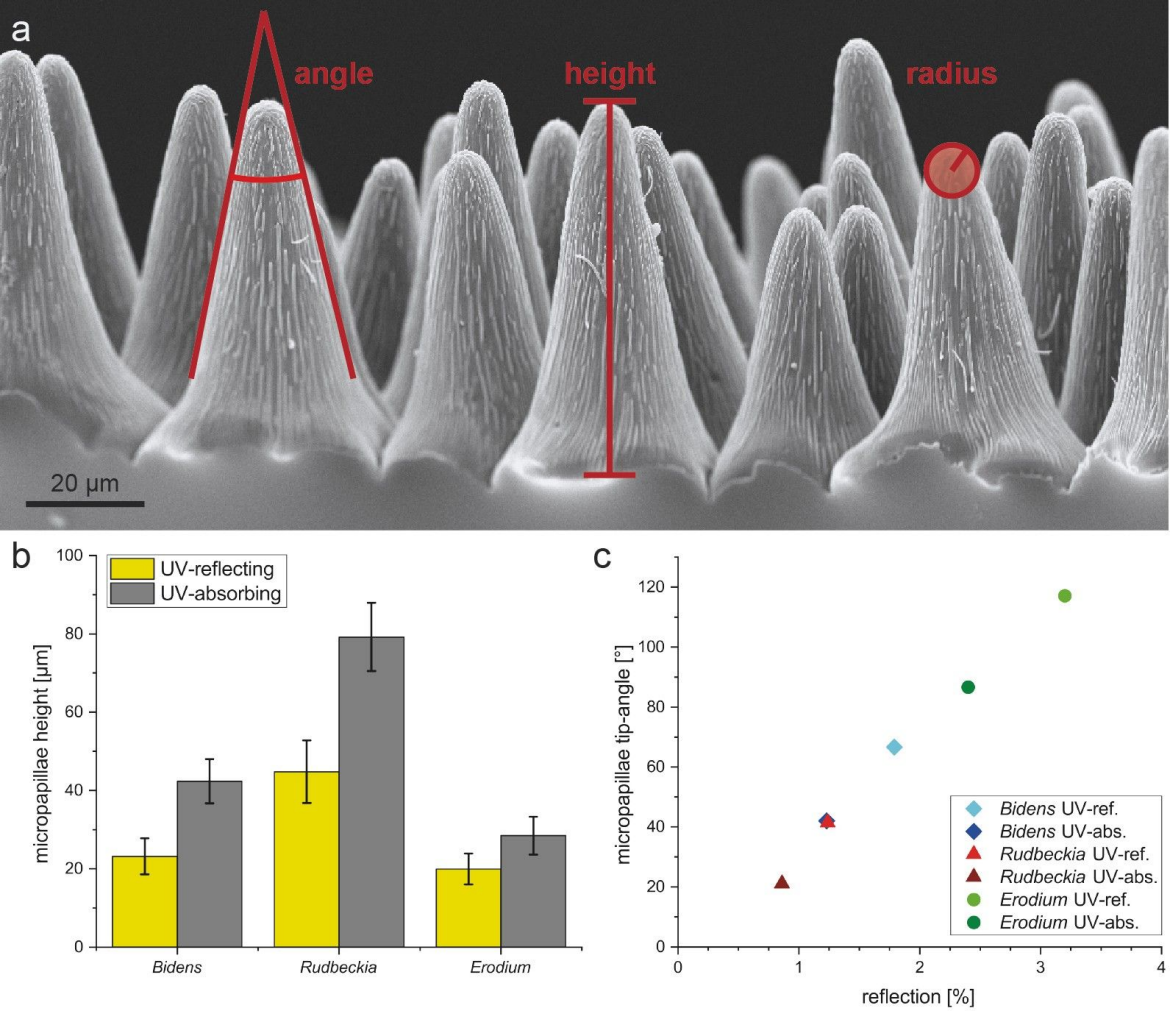
a) An SEM image with definitions showing the measurement principle of the parameters tip radius, papillae height, and tip angle. b) Comparison of the average (*N* = 50) height of the papillae of the UV-reflecting (yellow) and UV-absorbing (gray) areas of the petals. The black lines show the standard deviation. It can be seen that in each case the papillae in the absorbing area are much higher (at least 1.5 times) than in the reflecting ones. b) Tip angles of the papillae and surface reflections (for measurement details see Experimental section) of all six samples. It can be seen that the tip angle plays an important role. The higher the tip angle, the higher the reflection.

We analyzed the parameters of the epidermal cells in UV-absorbing and UV-reflecting areas (sample size *N* = 50). Figure 3 comprises the data of the papillae height (a) and the papillae tip angle (b). The papillae in the UV-absorbing areas of *B. ferulifolia* (42.4 ± 5.7 µm) and *R. fulgida* (79.9 ± 8.7 µm) are about twice as high as those in the related UV-reflecting areas (*B. ferulifolia* (23.2 ± 4.6 µm); *R. fulgida* (44.8 ± 8.0 µm)). The papillae in the UV-absorbing area (28.5 ± 4.9 µm) of *E. manescavii* are about one third higher than those in the UV-reflecting area (20.0 ± 4.0 µm). The average papillae height at the different absorbing and reflecting areas of the petals is shown in Figure 3.

**Figure 4:**
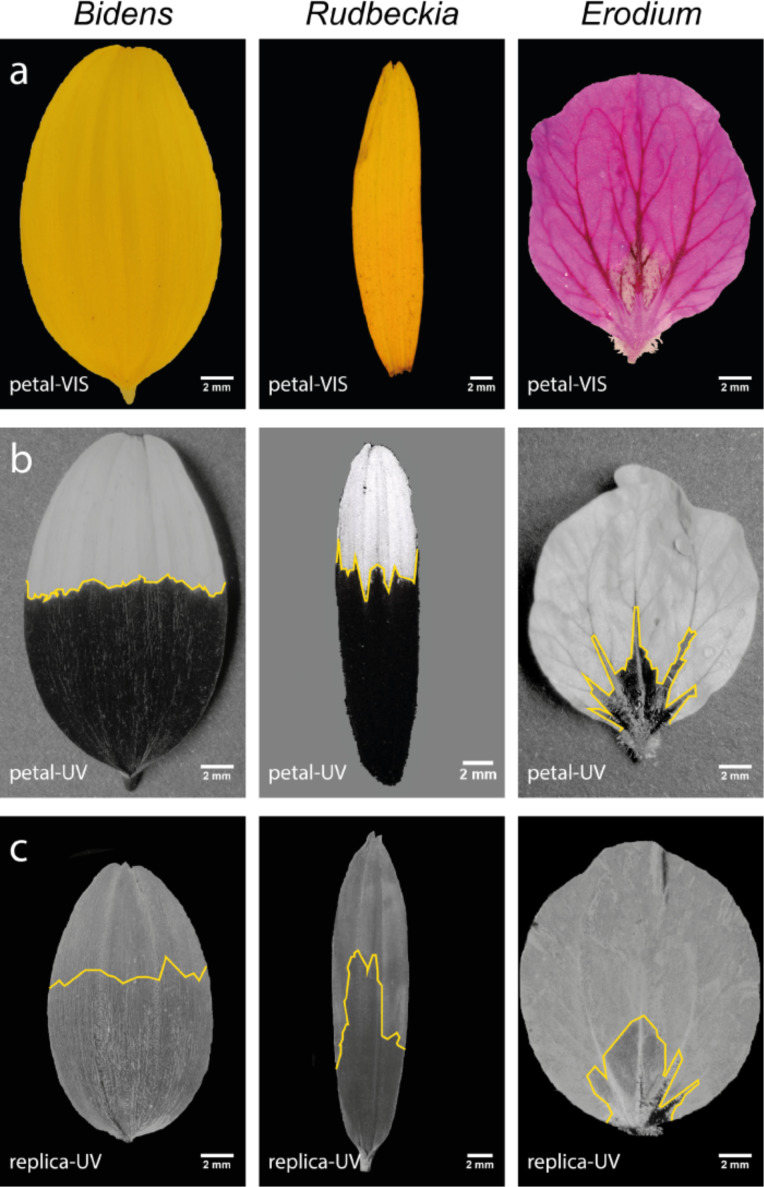
Petals and their polymer replicas of *B. ferulifolia* (left), *R. fulgida* (center), and *E. manescavii* (right). The petals are displayed in the VIS-spectrum (a) and the UV-spectrum (b). In the polymer replicas of the petals (c) the UV-patterns are present as well.

For the analysis of the UV-patterns, the petal surfaces of the three model species have been replicated. Comparison of the images of the petals with those of the replicas shows the quality of the replication process. In Figure 4, a comparison of the petals with their replicas, both in the VIS- and the UV-range, is presented. The petals show strong UV-patterns. It can be seen that the pictures of the replicas (Figure 4c) show almost the same UV-patterns but have less contrast. These pictures support the thesis that not only the pigments are responsible for the formation of the UV-patterns, but also the structure. In combination with the structural parameters it seems that the papillae height is also related to the UV-pattern. The higher the papillae, the more UV-radiation that reaches the petal, which supports the UV-pattern as more UV-radiation reaches the pigments inside the tissue. Further, it can be seen that the UV-patterns do not exactly match the petal UV-patterns. This leads to the assumption that the distribution of the pigments does not exactly match the regions with higher papillae.

**Figure 5:**
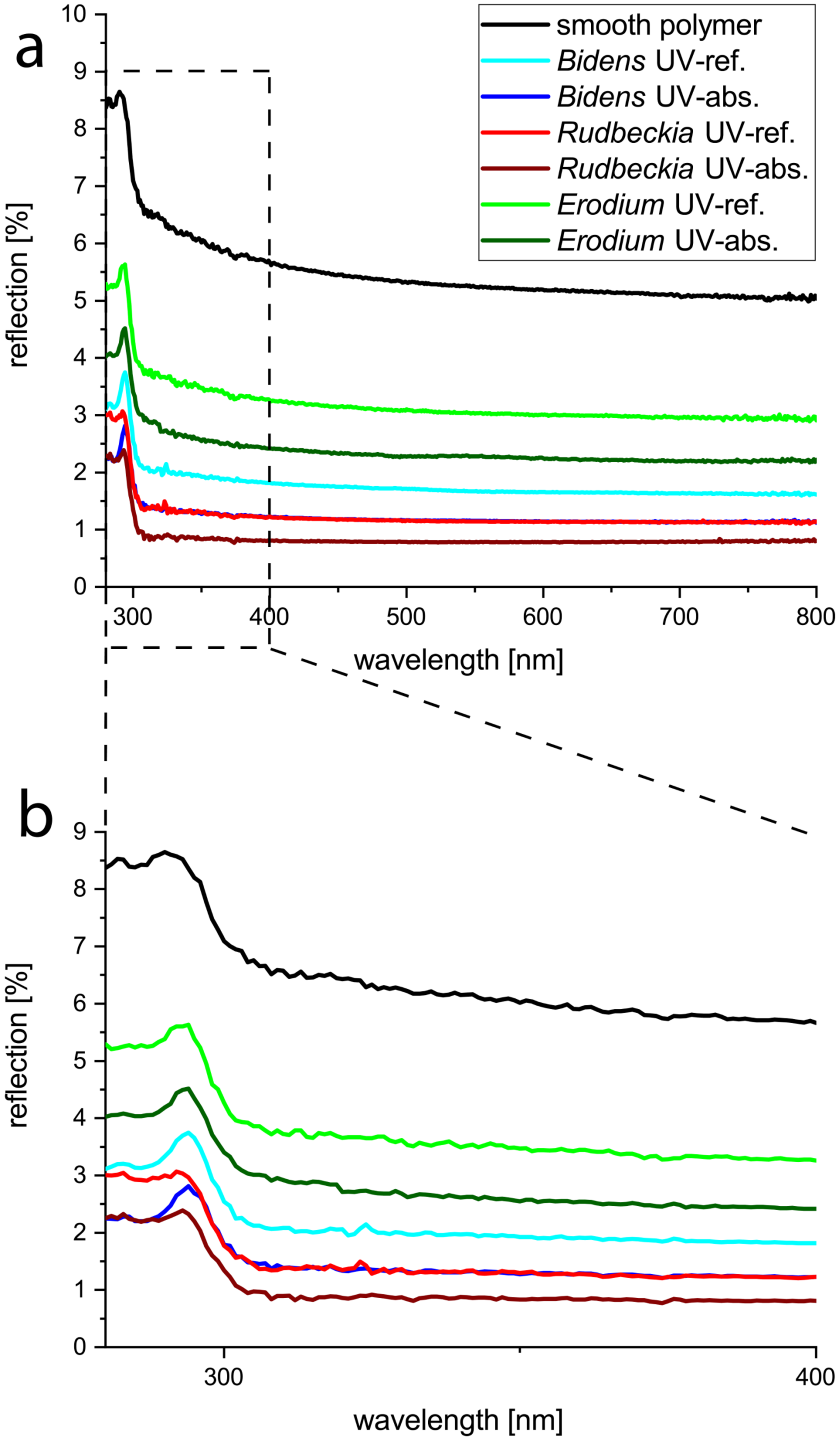
Percent reflection of light by polymer replicas of the petal surfaces at different wavelengths. As a reference, a smooth sample of the same polymer was also measured. a) Results measured in a wavelength range from 280 to 800 nm. b) More detailed view of the results measured between 280 and 400 nm.

Further, we investigated and quantified the influence of the surface structure on the appearance of the UV-patterns and compared the reflectivity of the UV-absorbing and the UV-reflecting areas within one petal as well as between the three model flowers. For this investigation, spectral measurements were performed as described in the Experimental section. As a reference, a smooth surface made of the replica polymer was used. These experiments showed that the structured replicas possess significantly lower reflection values than the comparable smooth reference. For better comparability, the reflection values at a wavelength of 366 nm were considered, as this wavelength was provided by the UV-lamp used for the UV-photography in the laboratory as well. While the smooth reference reflects about 5.8% (*r* = 5.8%) of the incoming light, the highest reflection value of the structured replicas was demonstrated by the replica of the UV-reflecting area of *E. manescavii* (*r* = 3.5%). The replica of the UV-absorbing area of *R. fulgida* (*r* = 0.8%) shows the lowest reflection. The reduction of the reflection by a papillose surface structure can be seen through the entire range of wavelengths from 280 nm to 800 nm measured in this experiment, i.e., from the UV–VIS-spectrum. The results are shown in Figure 5. We focused only on the UV-regime as further experiments would be needed to analyze possible patterns in the VIS-regime, which up to now could not be seen in the images we took. The significantly higher reflection values in the range between 280 nm and 400 nm are due to the material used for the replicas. As the differences between two samples are constant over the entire range from 280 nm to 800 nm, the material property can be assumed to have no influence on the measurements. The results of this experiment support the suggestions of Exner and Exner et al. [26] that the structure supports the amount of transmitted electromagnetic radiation into the petals. They also support the assumption that the transmission of UV-light into the petal and replica is also supported by the surface structure. Therefore, even if the pigments essentially cause the UV-patterns in petals, the surface structure at the petal adaxial surface also has a clear impact. Especially papillose-shaped epidermal cells significantly reduce the reflection of UV-light at the adaxial surfaces and support the absorption of UV-light. This means, in any case, more of the UV-radiation penetrates the surface and can reach the UV-pigments inside the petal tissue.

A more detailed comparison of the reflection values with the individual surface structure of the particular replicas shows that the higher the papillae, the lower the reflection values. For example, this trend is observed in the reflection values of *E. manescavii* as the papillae of the reflective area are about 20.0 ± 4.0 µm in height with *r* = 3.5%. In contrast, the papillae of the absorbing area are 28.0 ± 4.9 µm in height with a reflection of about 2.6%. However, not only the height of the papillae has an influence on the reflection values. The results show that a small tip angle of the papillae and cuticular foldings may also reduce the reflection. This could be seen, for example, when comparing the results of the reflecting areas of *B. ferulifolia* (height 23.0 µm, with cuticular folds; reflection of *r* = 2.0%) with the reflecting area of *E. manescavii* (height 20.0 µm, without cuticular folds; *r* = 3.7%). Although the height difference is very low, the reflection value of *E. manescavii* is almost twice as high as that of *B. ferulifolia*. This is even more evident as the tip angle of *E. manescavii* is smaller than that of *B. ferulifolia,* which should increase the absorption as seen in Figure 5. This result led to the assumption that cuticular foldings additionally increase the absorption of light.

## Conclusion

In this study we provided experimental data relating the structural difference of the UV-reflecting and UV-absorbing areas within flower petals. Furthermore, we provided the first quantitative measurements of these structural differences and their influence on the reflectivity and the scattering properties of light on the petal surface. We showed that the structures in the UV-absorbing area are higher in all three model plants and that they possess smaller tip angles than in the UV-reflecting areas. Using petal replicas, we separated this surface architecture from the cell pigments and thereby eliminated their influence on the UV-patterns. This procedure provides the first proof that the UV-patterns in flowers are not just pigment-based, but also structurally determined. Through this, differences in UV-reflectivity between the absorbing and reflecting areas of almost 50% have been found, which is new for the UV-regime. These results are important from a biological point of view as they are the first debatable measurements of the structural influence on UV-patterns and they show strong differences even within just the three model plants. From a technical point of view, these results are promising and important for different types of applications given that the optical properties of surfaces are becoming more relevant, for example, for hierarchically structured solar panels, biomimetic antireflective or absorbing coatings, lenses and many more.

## Experimental

### Plant material

Based on prior examinations of UV-reflection in some 8,000 species, three model species with distinct patterns were selected. Two are yellow flowering North American members of the sunflower family (Asteraceae): the Apache beggartick *Bidens ferulifolia* (Jacq.) Sweet. (Acc. No. 3428) and the Orange Coneflower *Rudbeckia fulgida var. sullivanii* (C.L. Boyton & Beadle) Cronquist (Acc. No. 29613). The third species from the Geranium family (Geraniaceae) is the purple storkbill from the Pyrenees *Erodium manescavii* (Coss., Acc. No. 3785). All were cultivated in the Botanical Gardens of the University of Bonn (the accession number (Acc. No.) is given in brackets).

### Replication technique

The petal surfaces were replicated using a two-step molding process described in detail by Koch et al. [39]. In this technique, first, a negative mold is generated followed by a positive. For the generation of the negative, the master (biological sample) is molded with a polyvinylsiloxane dental wax (President light body gel, ISO 4823, PLB; Coltene Whaldent, Hamburg, Germany). In the second step, these molds are filled with a two-component injection resin (RECKLI Injektionsharz EP, RECKLI GmbH, Herne, Germany).

### Scanning electron microscopy (SEM)

Images were recorded using a Cambridge Stereoscan 200 SEM (Zeiss GmbH, Oberkochen, Germany). A digital image processing system (DISS 5, Version 5.4.17.0, Point Electronic GmbH, Halle, Germany) was used to visualize and measure the surface structures of the petals. Prior to SEM examination, fresh plant material was critical point dried (CPD 020, Balzers Union, Balzers-Pfeifer GmbH, Aßlar, Germany). All samples were sputter-coated with a 30 nm gold layer (Balzers Union SCD 040, Balzers-Pfeifer GmbH, Aßlar, Germany). The stable replicas did not require special preparation. To examine the height, the mid-width and tip diameter of the surface structures, and the freeze fractures of the petals were used.

### Ultraviolet photography

A Nikon D 300s digital camera was used to take photos under UV-lamp (Camag, Muttenz, Switzerland) illumination at 366 nm. An extension of the sensor detection spectrum of the D 300s camera from 340 nm to 1100 nm was implemented by Optik Makario (Mönchengladbach, Germany) and allowed images in the UV-spectrum to be captured. An analog Nikon FM2 was used to take pictures under environmental conditions. Both cameras were equipped with a UV-Nikkor 105 mm quartz objective (Nikon), an IR-neutralization filter (NG 52D, Optik Makario, Mönchengladbach, Germany) and a UV-transmission filter (SP2 400UV 52D, Optik Makario, Mönchengladbach, Deutschland) to eliminate wavelengths in the infrared and visible spectrum.

### Spectral reflectance measurements using a spectrometer with an integrating sphere

Diffuse reflectance measurements were acquired throughout the 280 nm to 800 nm spectrum using a commercially available double-beam spectrometer (Lambda 1050, Perkin Elmer, Massachusets, USA) with an internal integrating sphere (Labsphere RSA-PE-20, 600 mm), whereby the total reflectance (*r*) was measured. The reference beam irradiates a Spectralon™ surface, which was calibrated as a 100% (*r* = 100%) reflective surface. The samples were attached to an opening in the surface of the integrating sphere on the opposite side of the opening where the light source was situated. The polymer replicas were colored black using black dye and a thin layer of carbon black was embedded at the backside of the replicas. Both steps were undertaken to avoid reflection at the backside of the replicas and backscattered light. This ensures that only the light reflected at the structured surface was taken into account. In all cases our measurement showed significantly higher values in the wavelength range between 280 nm and 300 nm. These higher values are likely due to autofluorescence of the material. But as all replicas were made of the same material we did not consider this point.
